# A PUBLIC HEALTH APPROACH TO ALZHEIMER’S DISEASE RISK REDUCTION: WHERE WE ARE AND WHERE WE’RE HEADED

**DOI:** 10.1093/geroni/igac059.509

**Published:** 2022-12-20

**Authors:** John Omura, Eva Jackson, Kelly O'Brien

## Abstract

A growing body of evidence has identified potential modifiable risk factors for Alzheimer’s disease and related dementias (ADRD). In 2021, the National Plan to Address Alzheimer's Disease (National Plan) included a new goal to promote healthy aging and address risk factors to help delay onset or slow progression of ADRD. Applying a robust public health approach to ADRD risk reduction can help achieve meaningful progress at the population level. The activities outlined in the Building Our Largest Dementia (BOLD) Infrastructure for Alzheimer’s Act (P.L. 115-406) are designed to create a uniform national public health infrastructure with a focus on various issues including risk reduction. The purpose of this session is to illustrate a public health approach to ADRD risk reduction, including its current status along with future directions and priorities. An overview of the National Plan’s new goal regarding ADRD risk reduction (McGuire) and data highlighting the current burden of key modifiable risk factors in the United States along with important disparities (Omura) will be presented. Holt will describe how ADRD risk reduction is integrated into the work of BOLD funding recipients, and Head will present experiences implementing public health activities that support ADRD risk reduction in the field along with successes and lessons learned. Finally, priorities and future directions for a public health approach to ADRD risk reduction will be presented from the perspective of the CDC’s Building Our Largest Dementia Infrastructure Public Health Center of Excellence on Dementia Risk Reduction (Baumgart).

